# STAT-1 gain-of-function CMC: Remission of Oral Candidiasis during PD-1 Inhibitor Treatment of Oral Cancer

**DOI:** 10.1007/s10875-025-01948-1

**Published:** 2025-11-18

**Authors:** Jannik Helweg-Larsen, Ditte Marie L. Saunte, Line Borgwardt, Claus Andrup Kristensen, Hanne Marquart

**Affiliations:** 1 Department of Infectious Diseases, Copenhagen University Hospital, Rigshospitalet, Denmark; 2 Department of Allergy and Dermatology, Copenhagen University Hospitals, Gentofte, Denmark; 3 Department of Clinical Medicine, Faculty of Health and Medical Sciences, University of Copenhagen, Copenhagen, Denmark; 4 Department of Dermatology, Zealand University Hospital, Roskilde, Denmark; 5 Center of Genomic Medicine, Copenhagen University Hospital, Rigshospitalet, Denmark; 6 Department of Oncology, Copenhagen University Hospital, Rigshospitalet, Denmark; 7 Department of Clinical Immunology, Copenhagen University Hospital, Rigshospitalet, Denmark

To the Editor,

Heterozygous *STAT1* gain of function (GOF) variants in patients with chronic mucocutaneous candidiasis (CMC) are associated with PD-L1 overexpression and diminished TH17 activity.

Here, we present a 65-year-old woman with a *STAT1* GOF variant and severe CMC, who during treatment with anti-PD1 treatment for progressive head and neck cancer had complete regression of severe oral candidiasis.

## Case Story

Beginning in infancy, the patient had chronic oral candidiasis and onychomycosis, with the subsequent development of alopecia and thyroiditis. Frequent anti-fungal treatments were necessary.

At the age of 46 she was diagnosed with a squamous cell carcinoma (SCC) of the tongue, treated with partial resection of the tongue and radiotherapy. During the following years chronic oral candidiasis, caused by *Candida albicans*, worsened, with development of progressive pan-azole resistance. Local treatment with amphotericin B had some effect, but prolonged i.v. echinocandin treatments became increasingly necessary.

By the age of 36, a heterogeneous variant in *STAT1 (*NM_007315.4): c.1204_1205delinsTT, p.(Ala402Phe) was identified by sanger sequencing and years later verified by exome sequencing. Functional analyses verified delayed dephosphorylation of STAT1 after stimulation with IFNg (data unpublished, abstract presented on ESID meeting 2012). Subsequently by co-segregation analysis, the *STAT1* variant was identified in two daughters and three descendants with CMC. In agreement with Liu et al., the *STAT1* variant was classified as pathogenic after demonstration of reduced dephosphorylation [1].

Following several case reports of successful JAK inhibitor treatment for STAT1-GOF CMC, baricitinib therapy was attempted at the age of 63. However, during a three-month and subsequent one-month trial, no effect against oral candidiasis was observed. Instead, she experienced severe adverse effects, including increased stomatitis, nausea and pneumonia.

Over the following year, severe aphthous and gingival ulcerations worsened. Following tooth extractions, an oro-antral fistula developed from which biopsies taken during surgical revision revealed severe inflammation with hyphae. In the subsequent months, extensive necrotic gingival ulcerations appeared on the maxilla, suspected to be osteoradionecrosis secondary to previous radiotherapy. Due to the necrosis, extensive resection of necrotic bone was done, including a partial maxillectomy with reconstruction of the post-surgical defect using a temporalis muscle flap. However, post-operatively, in addition to necrosis and hyphae, SCC was detected in the maxillary bone. Only two months after the maxillectomy, recurrence of SCC was observed. Electrochemotherapy with bleomycin was applied to tumours in the hard palate and the lower lip. Two months later, another recurrence developed in the right maxillary sinus and the left side of the nose.

A high level of PD-L1 expression, with a combined positive score (CPS) of 70, was detected in SCC tumour samples, indicating a potential benefit from PD-1 inhibitor combination therapy. However, concerns were raised regarding the possibility of anti-PD-1 treatment exacerbating the patient’s extensive and severe oral candidiasis.

One year prior to the SCC diagnosis and immediately before pembrolizumab treatment, immunophenotyping demonstrated a high percentage of PD-1-positive CD4 + T-cells (90%) and CD8 + T-cells (58%).

Treatment with a PD-1 inhibitor was ultimately deemed appropriate, and pembrolizumab 200 mg every three weeks was initiated. Remarkably, shortly after commencing pembrolizumab, the oral candidiasis regressed significantly. Throughout the PD-1 inhibitor treatment, she experienced complete resolution of the oral candidiasis, confirmed by repeated negative oral cultures for *Candida*, without need of antifungal treatment.

Two months after initiating treatment, a drastic decrease in PD-1 expression on both CD4 + and CD8 + T-cells was observed, indicating near-complete receptor saturation by pembrolizumab using an antibody for flow analyses with same specificity as pembrolizumab. Three months later, the total T-cell count had declined, with a severe reduction in CD4 + T-cells and a significant decrease in CD8 + T-cells, which remained within the normal range. PD-1 expression on both CD4 + and CD8 + T-cells remained largely unchanged, consistent with continued near-complete receptor saturation.

The initial evaluation, conducted three to six months into treatment, revealed a mixed response. Palliative radiotherapy was administered to the progressing tumor in the left maxillary sinus and nasal cavity involving the skin on the upper lip and cheek. However, following a total of 15 cycles of pembrolizumab, the tumor progressed, with invasion through the skin. Chemotherapy (three cycles of paclitaxel and capecitabine) had no effect and there was rapid progressive, disfiguring, and aggressive ulcerative progression, leading to extensive open destruction of her maxilla.

Despite chemotherapy, radiotherapy and several episodes of bronchiectasis related pneumonia, requiring broad-spectrum antibiotic therapy she experienced no relapse of the previous severe oral candidiasis during treatment with pembrolizumab.

Following the cessation of pembrolizumab, shortly before the patient’s death, the total T-cell count normalised. However, the CD4 + T-cell count remained reduced although slightly increased, while the CD8 + T-cell count remained within the normal range. Notably, PD-1 expression on both CD4 + and CD8 + T-cells was significantly elevated, returning to levels observed before pembrolizumab treatment.

She was transferred to palliative care and died a few months later.

## Discussion

This case demonstrates the unexpected resolution of severe, chronic oral candidiasis in a patient with CMC during treatment of progressive head and neck cancer with pembrolizumab, suggesting a potential, though uncharacterized, beneficial effect of PD-1 blockade in STAT1 GOF CMC.

CMC can be caused by inborn errors of IL-17 immunity [2]. STAT1 GOF variants are associated with increased PD-L1 expression, which suppresses Th17 differentiation, a key immune response against *Candida* infections [3]. This suppression is mediated by IL-27-induced STAT1 activation, which enhances PD-L1 expression, leading to a deficiency in Th17 cell responses. Studies have demonstrated that PD-L1 blockade can partially restore Th17 differentiation in patients with STAT1 GOF mutations [4].

Our patient’s response to pembrolizumab, a PD-1 inhibitor, aligns with preclinical models suggesting that immune checkpoint inhibitors (ICIs) may enhance antifungal immunity by restoring T-cell function and cytokine production [5]. Shortly after initiating pembrolizumab, we observed a drastic decrease in PD-1 expression on CD4 + and CD8 + T-cells, followed by a reduction in total T-cell counts, which could possibly be consistent with ICI-induced T-cell activation and subsequent immune reprogramming.

While JAK inhibitors are now considered a first-line treatment for *STAT1* GOF-associated CMC [6] our patient did not respond to baricitinib and experienced significant adverse effects. This highlights the variability in treatment response and the potential for adverse effects associated with JAK inhibitors.

In the absence of direct characterization of Th17 function, a specific cause for the observed response remains speculative. However, a plausible hypothesis is that pembrolizumab may have restored sufficient TH-17 mediated antifungal immunity by overcoming a specific PD-L1 block induced by the STAT1 GOF mutation.

## Data Availability

No datasets were generated or analysed during the current study.

## Declarations

the patient and family.
